# The Genetic Contribution of West-African Ancestry to Protection against Central Obesity in African-American Men but Not Women: Results from the ARIC and MESA Studies

**DOI:** 10.3389/fgene.2016.00089

**Published:** 2016-06-01

**Authors:** Yann C. Klimentidis, Amit Arora, Jin Zhou, Rick Kittles, David B. Allison

**Affiliations:** ^1^Department of Epidemiology and Biostatistics, Mel and Enid Zuckerman College of Public Health, University of ArizonaTucson, AZ, USA; ^2^Department of Surgery, College of Medicine, University of ArizonaTucson, AZ, USA; ^3^Nutrition and Obesity Research Center, University of Alabama at BirminghamBirmingham, AL, USA; ^4^Office of Energetics, University of Alabama at BirminghamBirmingham, AL, USA

**Keywords:** African-American, genetic admixture, genetic ancestry, obesity, central obesity, waist-to-hip ratio, health disparities, sex differences

## Abstract

Over 80% of African-American (AA) women are overweight or obese. A large racial disparity between AA and European-Americans (EA) in obesity rates exists among women, but curiously not among men. Although socio-economic and/or cultural factors may partly account for this race-by-sex interaction, the potential involvement of genetic factors has not yet been investigated. Among 2814 self-identified AA in the Atherosclerosis Risk in Communities study, we estimated each individual's degree of West-African genetic ancestry using 3437 ancestry informative markers. We then tested whether sex modifies the association between West-African genetic ancestry and body mass index (BMI), waist-circumference (WC), and waist-to-hip ratio (WHR), adjusting for income and education levels, and examined associations of ancestry with the phenotypes separately in males and females. We replicated our findings in the Multi-Ethnic Study of Atherosclerosis (*n* = 1611 AA). In both studies, we find that West-African ancestry is negatively associated with obesity, especially central obesity, among AA men, but not among AA women (p_interaction_ = 4.14 × 10^−5^ in pooled analysis of WHR). In conclusion, our results suggest that the combination of male gender and West-African genetic ancestry is associated with protection against central adiposity, and suggest that the large racial disparity that exists among women, but not men, may be at least partly attributed to genetic factors.

## Introduction

In the United States, African Americans (AA) exhibit higher rates of overweight and obesity. However, this trend appears to be mainly driven by very high rates among AA women. According to data from the Centers for Disease Control and the National Center for Health Statistics, the proportions of overweight or obese AA and EA women are 82.2 and 60.9%, respectively, whereas for men, the proportions are 70.7 and 73.2%, respectively (CDC/NCHS, 2013). A similar trend exists for rates of type-2 diabetes, whereby the race disparity is larger among women than among men (Brancati et al., 2000; Signorello et al., 2007). This puzzling pattern of a greater racial disparity only among women for obesity has not yet been explored from a genetic perspective.

Previous studies have shown that West-African ancestry (WAA) is associated with a greater body mass index (BMI) (Fernandez et al., 2003; Tang et al., 2006; Halder et al., 2012; Nassir et al., 2012), and greater whole-body and trunk fat mass (Ochs-Balcom et al., 2013). On the other hand, several studies have shown that WAA and AA race are associated with a lower body fat percentage, including visceral fat (Yanovski et al., 1996; Gower et al., 1999; Beasley et al., 2009; Cardel et al., 2011). However, some of these studies examined only women, or only BMI, or did not examine sex differences in the association between WAA and obesity phenotypes. Therefore, little is known as to whether this pattern differs among men and women, potentially resulting in the striking racial disparity observed among women, but not among men. A recent study by Goonesekera et al. (2015) reported a stronger (positive) association between WAA and BMI, percent body fat, and waist circumference (WC) among women compared to men in a cohort study of Boston residents, and a stronger positive association between WAA and BMI among less educated women. These findings are consistent with the epidemiological pattern described above, and deserving of further examination.

Based on these previous studies, it is possible that WAA, representing a collection of many individual genetic variants, predisposes to less central/visceral adiposity, and that sex modifies this relationship through biological and/or environmental factors. To help further understand if genetic factors may play a role in the large racial disparity among women, and the relatively small racial disparity among men, we examined the interaction of WAA with sex on BMI, WC, and waist-to-hip ratio (WHR) in two cohorts of AA. We also examined whether the association of WAA with these phenotypes differs according to income, education, or menopausal status.

## Methods

### Studies

We used data from the Atherosclerosis Risk in Communities (ARIC) study (The Aric Investigators, 1989) and from the Multi-Ethnic Study of Atherosclerosis (MESA) study (Bild et al., 2002) (*n* = 2814 and 1611 AA, respectively). Briefly, ARIC is a prospective multi-center cohort study of EA and AA, aged 45–64, to examine risk factors for atherosclerosis. The MESA study is also a prospective multi-center cohort study of men and women, aged 45–85, of diverse ethnic and racial backgrounds, to investigate the risk factors for cardiovascular disease. We only included self-identified AA in this study. Datasets were obtained from the database of Genotypes and Phenotypes (dbGaP). We obtained IRB approval from the University of Arizona.

### Genotypes and ancestry estimation

Both ARIC and MESA participants were genotyped with the Affymetrix Genome-Wide Human SNP Array 6.0 (Affymetrix, Santa Clara, CA, USA). To estimate WAA, we used a panel of 3437 SNPs developed by Tandon et al. which are especially informative for West African vs. European ancestry among AA, are spaced throughout the genome, are not in linkage disequilibrium with each other in the ancestral populations, and are available on the Affymetrix 6.0 array (Tandon et al., 2011). With the genotypes of these SNPs, we performed a principal components analysis (PCA) on the scaled and centered genotypes, using the *prcomp* function in R Statistical Software (R Development Core Team, 2011). To confirm that PC1 of this analysis corresponds to WAA, we used a supervised Bayesian approach with STRUCTURE v2.3 software (Pritchard et al., 2000) with genotypes at the same set of SNPs for HapMap reference individuals (Yoruba in Ibadan, Nigeria: *n* = 176, and Utah residents with ancestry from northern and western Europe: *n* = 174) (The International HapMap Consortium, 2003) as putative W. African and European parental populations. We used *K* = 2, representing two founding populations, and a burn-in length of 20,000 for 80,000 repetitions. There was a very strong relationship between PC1 and WAA as determined by the supervised ancestry analysis (*r*^2^≥ 0.96).

### Phenotypes, income, education, and menopausal status

BMI was calculated as weight (kg) divided by squared height (m^2^). Waist measurements were taken at the umbilicus to the nearest 0.1 cm in MESA, and nearest 1 cm in ARIC. Hip circumference was measured from the largest diameter of the hip, to the nearest 0.1 cm in MESA, and nearest 1 cm in ARIC. WHR was calculated by dividing waist circumference by hip circumference. Income was recoded to correspond to yearly household/family income in US dollars. As yearly income intervals were presented in the questionnaires, we chose midpoints of these intervals. In both datasets, education was recoded to correspond to years of education (using interval midpoints, if needed). Menopausal status was assessed as a binary variable in both cohorts using the response to a question asking if women had gone through menopause or not.

### Statistical analyses

Differences in WAA, phenotypes, and other characteristics were compared across cohorts and in men vs. women using *t*-tests. The difference in percentage of women having undergone menopause between the two cohorts was tested using a chi-squared test. We examined the distributions of these three phenotypes as well as the distributions of WAA, income, and education to assess normality. We then used linear regression to test the association of WAA with each phenotype among all, male, and female AA, in each study and in the combined dataset. We log transformed BMI and WC to conform to the assumptions of residual normality. We included age, sex, income, education, and WAA as covariates. In the combined analyses, we additionally included a two-level categorical “study” variable (ARIC/MESA) in order to account for variation across studies. The interaction of WAA with sex was modeled as the product of these two variables and included as a predictor in the linear regression model. To test whether the association of WAA with the obesity-related phenotypes differs according to income, education or menopausal status, we tested the interaction of WAA with each phenotype separately in men and/or women, including in the model all other covariates. Statistical analyses were carried out with R Statistical software, version 2.15.2 (R Development Core Team, 2011).

## Results

### Association of WAA with obesity- and metabolic-related phenotypes

**Figure 1:**
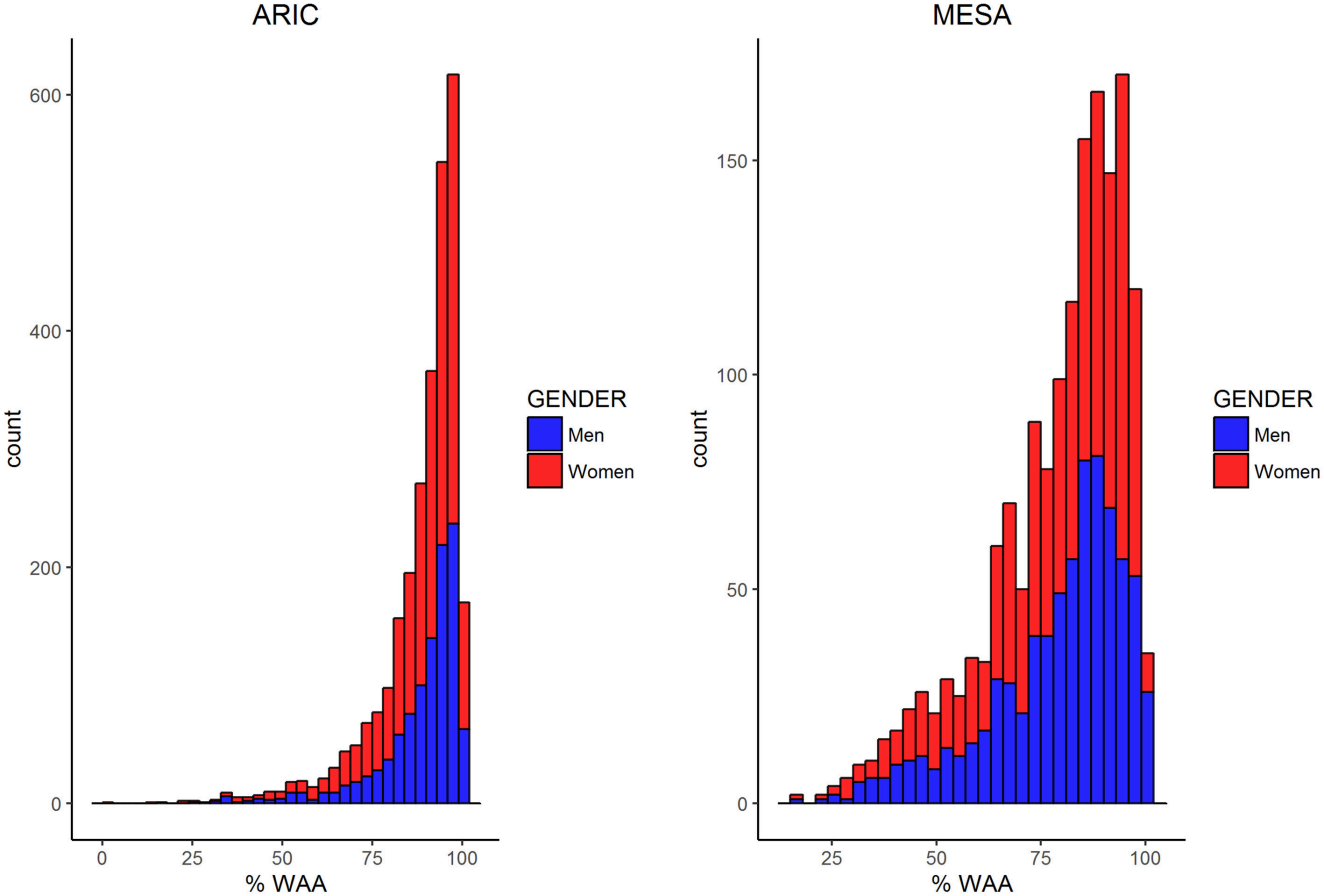
**Histograms comparing the distribution of WAA among men and women in ARIC and MESA**.

Characteristics of AA participants in MESA and ARIC are shown in Table 1. In addition to mean age being higher in MESA, levels of income, education, BMI, and WC are also higher in MESA. The distribution of WAA in both ARIC and MESA are shown in Figure 1. As shown in Table 1, WAA is significantly higher among ARIC participants (88%) than among MESA participants (80%). However, we do not find any difference in WAA between AA men and women in either MESA or ARIC (*p* = 0.81 and 0.93, respectively, see Figure 1). The main effect models for ARIC and MESA and for each of the three phenotypes are shown in Table 2. Notably, among AA in ARIC, WAA is positively and significantly associated with BMI in ARIC AA (*p* = 0.004), but not MESA AA (see Table 2). In general, we observe a trend of a positive association between WAA and BMI, no consistent association for WC, and a negative association of WAA with WHR. We also observe that both income and education are negatively associated with all three phenotypes, and these associations are much stronger in women, and nearly absent in men (data not shown).

**Table 1:** **Characteristics of male and female African-American participants in ARIC and MESA**.

	**ARIC**		**MESA**		***p*-value**
	**Men**	**Women**	**Men**	**Women**	
*n*	1078	1736	743	868	
Age (years)	53.8±6.0	53.2±5.7	62.4±10.2	62.2±10.0	2.07E-188
Education (years)	11.1±6.0	11.8±5.4	14.0±3.6	14.1±3.5	1.93E-70
Income ($K per year)	25.7±20.4	19.0±17.7	53.5±33.6	41.3±29.6	1.67E-143
BMI (kg/m^2^)	28.0±4.9	30.8±6.5	28.8±4.7	31.3±6.5	0.016
WC (cm)	97.9±12.8	100.3±16.2	100.8±12.8	101.7±16.2	1.75E-4
WHR	0.94±0.06	0.91±0.08	0.95±0.06	0.90±0.08	0.51
WAA%	88±12	88±12	80±16	79±17	6.85E-86
Menopause (%)		93.6		85.1	1.88E-8

*P-value in last column refers to comparison of entire two cohorts (men and women combined). The proportion of women having undergone menopause are based on 863 and 818 non-missing values in ARIC, and MESA, respectively*.

**Table 2:** **Main-effect models of association with anthropometric phenotypes**.

	**BMI**		**WC**		**WHR**	
	**β**	***p***	**β**	***p***	**β**	***p***
**ARIC**						
Age	−0.002	0.003	5.6E-04	0.292	0.002	5.0E-09
Sex	−0.087	4.0E-27	−0.021	0.001	0.037	3.1E-34
Income	−7.9E-05	0.738	−3.0E-04	0.100	−3.7E-04	3.9E-05
Education	−0.003	1.7E-04	−0.003	8.6E-06	−0.002	2.8E-08
WAA	0.095	0.004	0.026	0.297	−0.008	0.497
**MESA**						
Age	−0.003	9.9E-08	−9.8E-04	0.037	9.9E-04	5.3E-07
Sex	−0.078	4.8E-15	0.003	0.724	0.053	7.9E-42
Income	4.7E-05	0.791	−3.8E-04	0.017	−2.9E-04	3.1E-05
Education	−0.003	0.045	−0.002	0.188	−5.2E-04	0.380
WAA	0.010	0.737	−0.031	0.241	−0.027	0.018

**Figure 2:**
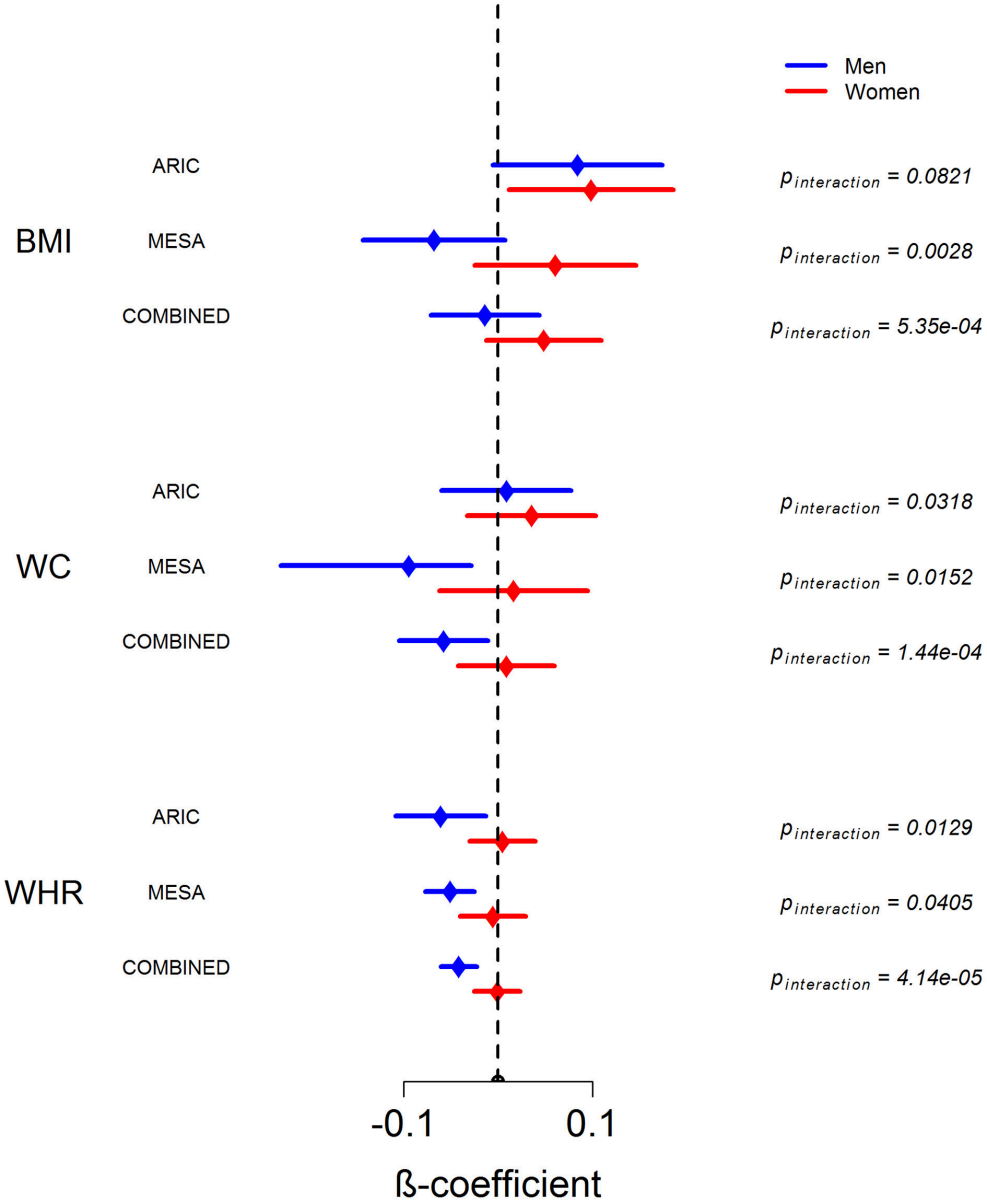
**Sex-stratified analysis of association between WAA and BMI, WC, and WHR in ARIC, MESA and in combined dataset**. Error bars represent 95% confidence intervals around the beta coefficients.

Figure 2 shows the sex-stratified analysis of WAA with BMI, WC, and WHR, as well as the respective interaction *p*-values for the interaction of sex with WAA. The interaction of sex with WAA is most pronounced for WHR (p_interaction_ = 4.14 × 10^−5^), whereby we observe a negative association of WAA with WHR among men. Among women, there is essentially no association between WAA and WHR. The interaction trend is similar but slightly weaker for WC (p_interaction_ = 1.44 × 10^−4^) and BMI (p_interaction_ = 5.35 × 10^−4^). Among women, there is a trend of a positive association of WAA with BMI and WC. Finally, we observed a trend in which the overall association of WAA with BMI and WC appears to be more positive in ARIC than in MESA, which is corroborated by results of the main-effects model shown in Table 2.

In the pooled dataset, we do not find any evidence for statistically significant interactions of WAA with either income or education among men or women, or of WAA with menopausal status among women (see Table 3).

**Table 3:** **Interaction of WAA with income, education, and menopausal status on three obesity-related phenotypes in men and/or women**.

	**BMI**		**WC**		**WHR**	
	**β_interaction_**	**p_interaction_**	**β_interaction_**	**p_interaction_**	**β_interaction_**	**p_interaction_**
**INCOME**						
Women	−0.0014	0.18	−0.0016	0.067	−0.00058	0.17
Men	0.0011	0.23	0.00042	0.58	0.00027	0.37
**EDUCATION**						
Women	0.013	0.06	0.0065	0.24	0.0016	0.54
Men	0.0025	0.66	0.0011	0.81	0.00085	0.66
**MENOPAUSE**						
Women	−0.147	0.25	−0.055	0.29	−0.042	0.70

## Discussion

We used genetic admixture/ancestry as a tool to investigate whether there may be a genetic basis to the large racial disparity in obesity that exists among women, but not among men. Our findings suggest that WAA is associated with protection against central obesity among AA men, but not among AA women. The pattern may explain to a great extent the much greater racial disparity among women (or alternatively, the much greater sex disparity among AA, as compared to EA). One interpretation is that AAs are exposed to environmental and/or cultural factors that predispose them to greater obesity than EAs. Possibly, some of the genes that are inherited as part of their West-African ancestry are protective against obesity, thereby “canceling out” the obesifying effects of environment/culture, but only in men. Another interpretation is that genetic protection is afforded to all individuals of African descent, but this protection is overwhelmed by cultural and/or other factors in women. For example, previous studies have found a tendency for a larger ideal body size among AA women as compared to White women (e.g., Rucker and Cash, 1992; Becker et al., 1999). In addition, evidence for greater obesity risk among women than men from socio-economic factors has been noted in a cross-country study (Wells et al., 2012), and in several studies examining gender and race gaps in obesity in the United States (Zhang and Wang, 2004; Robinson et al., 2009; Seamans et al., 2015).

Our findings are quite consistent across the two cohorts examined. However, we did observe a trend of a stronger positive association of WAA with BMI and WC in ARIC than in MESA in both genders. This difference could be attributed to differences in age, WAA, or other characteristics that differ between these two cohorts, as shown in Table 1. The pattern of stronger positive association among women however, holds up in both cohorts for all three phenotypes, and is quite consistent across both cohorts for WHR.

Previous studies have shown that AA have lower levels of central adiposity than EAs and other ethnic/racial groups (Yanovski et al., 1996; Beasley et al., 2009), and that WAA is associated with lower central adiposity (Cardel et al., 2011). However, some previous studies were limited to female participants, or did not examine sex differences in the association of WAA with obesity phenotypes. Here, we confirm the Goonesekera et al. finding that the association of WAA with BMI and WC is stronger among women. Our results suggest that this trend may be driven by the negative association of WAA with WC and WHR among men.

This study is strengthened by the use of two cohorts of AA with relatively large sample sizes, the use of multiple obesity-related measurements, and the availability of thousands of SNPs to more accurately estimate WAA. Limitations include the lack of more refined and metabolically relevant measurements such as amount of visceral adiposity, and the absence of other potentially important socio-economic confounders such as discrimination, and access to health-care. Regarding the latter limitation, it is difficult to tease genetic factors apart from environmental factors. Thus, it could be that variation in WAA is also capturing variation in environmental, social, cultural factors beyond those that we have included in our models as covariates.

This study suggests that there are specific genetic variants and physiological mechanism(s) that differ among West African and European populations, which when in the context of male sex, result in lower central adiposity. Further research, likely requiring larger sample sizes, is needed to identify these putative specific genetic factors and their corresponding physiological mechanisms. The identification and understanding of these mechanisms could lead to improved prevention and treatment approaches. It will also be important to also consider and examine non-genetic factors that may underlie the greater racial disparity among women.

## Author contributions

YK and DA contributed to the conception and design of this work. YK and AA analyzed the data. YK, JZ, RK, AA and DA contributed to the interpretation of the data, and to the writing and revising of the manuscript.

## Funding

YK and AA are supported by a National Institutes of Health (NIH) grant: K01DK095032. DA is supported by NIH grant P30DK056336. The opinions expressed are those of the authors and do not necessarily represent those of the NIH or any other organization.

### Conflict of interest statement

Dr. DA has received, anticipates, or has had financial interests with the Frontiers Foundation; University of Wisconsin; University of Arizona; Paul Weiss, Wharton and Garrison LLP; and Sage Publications. The remaining authors declare that the research was conducted in the absence of any commercial or financial relationships that could be construed as a potential conflict of interest.
